# Serology for Neosporosis, Q fever and Brucellosis to assess the cause of abortion in two dairy cattle herds in Ecuador

**DOI:** 10.1186/s12917-019-1924-7

**Published:** 2019-06-11

**Authors:** Darwin Changoluisa, Ismar A. Rivera-Olivero, Gustavo Echeverria, Miguel Angel Garcia-Bereguiain, Jacobus H. de Waard, the working group “Applied Microbiology” of the School of Biological Sciences and Engineering at Yachay Tech University, Sofía Abad-Sojos, María J. Aldáz-Villao, Estefanía Benavides, Carla M. Brito, Angie Changuan, Marlon Chimarro, Nicole Espinosa, Martha Galarraga, Marlon A. Gancino Guevara, Keila Gómez, Cristian Guzmán, Nigel F. Haro Sisa, Henry J. Herrera, Juan C. Laglaguano, Kerly Leon-Ordóñez, Katherine Loachamin, Karla Miño, Cristhian Preciado, Sebastián Rodríguez, María P. Romero, Karen Sánchez, Henry Secaira-Morocho, Carolina Serrano-Larrea, Jorge E. Simón-Tanquero, María E. Sulen, Odalys Torres, Paolo Vallejo-Janeta, Sabrina Yanez, Marlon Zambrano-Mila

**Affiliations:** 1 School of Biological Sciences and Engineering, Yachay Tech University, Urcuquí, Ecuador; 2 One Health Research Group, Facultad de Ciencias de la Salud, Universidad de las Américas, Quito, Ecuador; 3 Instituto de Investigacion en Salud Publica y Zoonosis, CIZ, Universidad Central del Ecuador, Quito, Ecuador; 4 The working group “Applied Microbiology”, School of Biological Sciences and Engineering, Yachay Tech University, Urcuquí, Ecuador

**Keywords:** Neosporosis, *Neospora*, Q fever, *Coxiella*, Brucellosis, *Brucella*, Cattle, Ecuador

## Abstract

**Background:**

Determining the infectious cause of abortion in cattle is difficult. This case-control study was set up to investigate the infectious causes of abortion by determining the seroprevalence of three reproductive pathogens in dairy cattle in Ecuador and their association with abortion: *Brucella abortus, Neospora caninum* and *Coxiella burnetii*.

**Results:**

Ninety-five blood samples were obtained from cows that had experienced a mid- or late gestation abortion of their first calf and seventy-seven samples from a control group of cows with the same age that did not experience abortion problems. No antibodies were detected for *B. abortus* in any of the serum samples, but a high seroprevalence for both *C. burnetii* (52.9%) and *N. caninum* infection (21.5%) was found in group of cows. The seroprevalence of *N. caninum* infection in cattle that had experienced abortions was significantly higher (*p* < 0.05) than the seroprevalence in the control cows on one of the cattle farms, but no association between abortion and seropositivity for *C. burnetii* was found.

**Conclusion:**

We conclude that Neosporosis plays an important role in the epidemiology of abortion on one cattle farm, but that Q fever is apparently not an important cause for abortion in this setting.

**Electronic supplementary material:**

The online version of this article (10.1186/s12917-019-1924-7) contains supplementary material, which is available to authorized users.

## Background

Abortion in cattle is defined as the premature expulsion of the fetus between day 50 and day 270 of gestation. Most cattle herds suffer an abortion rate of 1-2% and it has been suggested that an annual abortion rate up to 5% is considered normal [1]. In general, the percentage of abortion cases for which a definitive diagnosis is made is very low. For example, in Great Britain less than one third of abortion cases are submitted to the laboratory for diagnosis [1]. In developing countries, this percentage is probably much lower and people usually have no intention of seeking a diagnosis, rather cows that have aborted are culled.

Abortions cause significant economic loss, especially those occurring during the last stage of pregnancy. Estimates of the cost of an abortion to a producer range from $90 to $1900, depending on the gestation phase in which it occurs. A midterm abortion costs the producer between $600 and $1000 [2]. Costs include those associated with establishing the diagnosis, re-breeding cows that aborted, sperm or embryo costs, possible loss of milk yield and replacement costs if cows that have aborted are culled.

Determining the cause of abortion in cattle is difficult and a major challenge to the herd owner and veterinarian. Infectious agents represent the leading etiology and the majority of diagnosed abortions are attributed to infections with the bacteria *Brucella abortus* and *Leptospira interrogans*, the protozoa *Neospora caninum* and two viruses: Infectious Bovine Rhinotracheitis or bovine herpesvirus (IBR or BHV) and Bovine Viral Diarrhoea (BVD) [3]. Moreover, *Coxiella burnetii*, the causal agent of Q fever which is a zoonotic disease, has been related to stillbirth, aborted fetuses and the delivery of weak and nonviable neonates in ruminants. Yet, the correlation between *Coxiella* seropositivity and abortion risk in bovines is far less understood [4, 5].

Few reports concerning the infectious cause of abortion have been carried out in Latin America and this paper aims to assess the infectious agent that induces abortion in two dairy herds, each of about 2000 heads, from a tropical region of Ecuador. These cattle herds have an annual abortion rate of between 3 and 5%.

In a case-control study and using commercially available ELISAs, we determined the seroprevalence for Brucellosis, Neosporosis and Q fever in cattle that had experienced an abortion during mid- to late gestation. We compared this prevalence with the seroprevalence in a randomly-selected control group of cattle from the same cattle farms and of the same age that had never suffered an abortion and we determined if one of the aforementioned cattle diseases could be associated with abortion.

Brucellosis is endemic in Ecuador and studies have reported a seroprevalence of up to 17% and a herd prevalence of 45% [6]. Concerning the infection with *C. burnetii* in Ecuador, few data are available and a within-herd seroprevalence of more than 40% and a herd prevalence of 47% have been reported [7, 8]. Neosporosis has never been studied in this country, hence the prevalence of this infestation in cattle is unknown. All three diseases are on the World Organization of Animal Health’s (OIE) list of notifiable diseases. Ecuador has reported the presence of Brucellosis in its territory to the OIE, but no official reports concerning Q fever and Neosporosis have been emitted [9].

## Results

A total of 172 cows from two dairy farms- 93 cows from farm A and 79 cows from farm B - were tested with commercially available ELISAs for the presence of antibodies against Brucellosis, Q fever and Neosporosis. The results of the serodiagnosis are summarized in Table 1. In both cattle herds, no antibodies against *B. abortus* were detected and the results for Brucellosis testing were omitted from this table. The overall seroprevalence of Q fever and Neosporosis were 52.9 and 21.5%, respectively. The prevalence of Q fever was 49.4% in cows with a first-calf abortion and 57% in the control cows and that of Neosporosis, 28.4 and 13.0%, respectively. Mixed infections with *C. burnetii* and *N. caninum* were also common (*n* = 20 or 11.6%) with 14 (14.7%) mixed infections in the cows that had a history of abortion and 6 (7.8%) mixed infections in the control group.

**Table 1:** Serodiagnosis of Q fever and Neosporosis in two cattle farms in Ecuador. We tested cows with a history of a first-calf abortion (*n* = 95) and a control group of cows of the same age group that had never aborted (*n* = 77)

Cows tested	Cows that aborted Q fever +	Control cows Q fever +	Cows that aborted Neosporosis +	Control cows Neosporosis +
Farm A	34 (60.7%)	22 (59.5%)	21 (37.5%)	5 (13.5%)
Farm B	13 (33.3%)	22 (55.0%)	6 (15.4%)	5 (12.5%)
Total = 172	47 (49.4%)	44 (57.1%)	27 (28.4%)	10 (13.0%)

A statistical analysis of prevalence rates for the cows from both cattle farms showed a positive association between *N. caninum* infection and abortion (Odds Ratio [OR] 2.66; [CI95% 1. 19-5.92]; *p* = 0.014) while no significant association between *C. burnetii* infection and abortion was found (Odds Ratio [OR] 0.73; [CI95% 0.40–1.34]; *p* = 0.316). Furthermore, no association between the presence of both infections (Q fever and Neosporosis) and abortion was found (Odds Ratio [OR] 2.04; [CI95% 0.74–5.60]; *p* = 0.158).

When both farms were analyzed separately, on Farm A a positive association between *N. caninum* infection and abortion was found once more (Odds Ratio [OR] 3.84; [CI 95% 1.295 3- 11.3844]; *p* = 0.012), but on farm B no association between the presence of Q fever and/or Neosporosis and abortion was found (*p* > 0.05).

## Discussion

Relatively few studies have been performed in Latin America regarding the cause of abortion in cows and most studies come from Brazil. In Ecuador, the cause of abortion in cows is mostly unexplored. In South America, the most assessed infectious diseases as the cause of abortion are Neosporosis and Brucellosis. To the best of our knowledge, no reports exist on this continent evaluating the burden of Q-fever.

The aim of our study was to find an infectious cause for first-calf abortion in two cattle farms in a tropical part of Ecuador, both farms with a relatively ¨normal¨ abortion rate of 3-5%. This case-control study included 95 cows that had aborted once in the second or third stage of gestation and 77 aged-matched control cows without a history of abortion. In our study, we used commercial ELISA kits to determine the presence of antibodies against Brucellosis, Q fever or Neosporosis and we looked for an association with abortion. We did not search for antibodies against *Leptospira interrogans,* IBR or BVD. These two viruses and the five *Leptospira* serovars are commonly associated with abortion in cattle [10]. However, both farms vaccinate their cattle with the combination vaccine Cattlemaster®GoldFP®5 L5 against said viruses and the *Leptospira interrogans* serovars: grippotyphosa, pomona, hardjo, canicola and icterohaemorrhagiae. Vaccination-induced antibodies against these microorganisms are to be expected in all the cattle under study.

Concerning Brucellosis, although there is an ongoing vaccination program on both cattle farms with the vaccine strain RB51, we tested for Brucellosis antibodies because the disease is highly endemic in Ecuador. Moreover, we recently detected that a dog on one of the cattle farms was positive for *Brucella abortus*. (Personal communication JHdW, MG) and a previous study determined that the efficacy of the vaccination with RB51 is only partial, preventing approximately 60% of the cows and fetuses from infection [11]. We therefore cannot exclude infection with *B. abortus* as a cause of abortion, but no seropositive animals were detected in these cattle herds and thus we exclude abortion due to Brucellosis.

It is generally accepted that chronic infection with *C. burnetii* may cause abortion, premature birth, stillbirth or weak offspring in cattle, sheep and goats [12]. The infection rate we found on both cattle farms was high (49%), however in our study no association between Q fever and abortion could be established. In some publications, a strong association has been found between *C. burnetii* infection and abortion [13]. 22% [14] of abortion cases in a study in Italy and and 37% [15] of one in Cyprus were due to an infection with *C. burnetii.* In another study, seroprevalence for Q fever was found to be twice as high in cows that had aborted in comparison with an aged-matched control group [16]. However, our findings support the conclusions of a review on Q fever that *C. burnetii* infection is an infrequent cause of abortion in cattle [17].

*Neospora caninum* is considered one of the most frequent infectious organisms causing abortion in cattle worldwide [18, 19]. Concerning South America, a review of the year 2005 reports evidence of exposure to *N. caninum* in cattle, goats, sheep, dogs, cats, water buffaloes, alpacas, llamas, opossums, wolves and other wild canids from Argentina, Brazil, Chile, Paraguay, Peru and Uruguay [20].

Here we report, for the first time, the presence of Neosporosis in Ecuador and in this small study, with only 172 cows tested, we found an overall seroprevalence of 21.5%. Blood samples were taken between 2 and 10 months after abortion had taken place and the prevalence of Neosporosis could be even higher in the tested cattle herds because of fluctuations in antibody levels that seemed to be higher just before and after the abortion had taken place [21]. We show that the infection with this parasite on one farm increased the risk of an abortion by a factor of almost 4. However, on the other cattle farm, no association between seropositivity for *N. caninum* and abortion could be found. No sound explication is readily available to explain the differences between these two cattle farms. Nonetheless, the presence of different *N. caninum* strains on the two cattle farms, that differed in pathogenicity cannot be excluded [22]. In Spain, absence of fetal death has been reported in pregnant heifers inoculated with the NC-Spain-1- H isolate of *Neospora caninum*, an isolate of low virulence, whereas fetal death occurred in heifers inoculated with the control strain NC1 [23]. The presence of *N. caninum* strains with different virulence could also explains why the two farms in our study had a relatively low abortion rate of 3-5% in comparison with a relatively high *N. caninum* seroprevalence of 21.5%. Hence, more research is necessary and we have planned to genotype the Neospora strains of our study area. We will also look to directly confirm the presence of *N. caninum* in abortion material or in the fetuses, as was achieved in a study in southern Brazil, where PCR revealed that 38.8% of the aborted fetuses were positive for *N. caninum* [24].

## Conclusions

We conclude that more investigations, especially on molecular level and DNA analysis for infectious agents in abortion material, is necessary to confirm our observations. In addition, as infection is the most common cause of abortion in cattle, unusual causes should be included in future studies, like *Chlamydophila abortus*, *Salmonella dublin* or *Listeria monocytogenes* infections. In a case-control study, an association between *Chlamydia psittaci* seropositivity and abortion in Italian dairy cows has been shown [25] but the impact of this microorganism on abortion in South America has never been investigated. Concerning other infectious causes of abortion, bovine venereal diseases, trichomoniasis and genital campylobacteriosis (caused respectively by a protozoan parasite and a gram-negative bacteria) should be included in future studies, as all of them can cause reproductive failure, including repeated estrus, early embryonic death and abortions [26]. To the best of our knowledge, the presence of these diseases has never been studied in Ecuador.

### Limitations of this study

This is a small-scale retrospective study of a limited range of abortifacients on two dairy farms, the first of its kind in Ecuador. Budget constraints did not permit us to study other abortifacients mentioned in the conclusions. In this manuscript, we report that abortifacients, included in the combination vaccine Cattlemaster®GoldFP®5 L5, were not studied because we expected an antibody response. However, limited public information is available concerning the efficacy of the Cattlemaster vaccine. We therefore will include testing for Leptospirosis and BVD in future investigations and verify if in fact there is association of vaccination with serological results, as these are important causes of abortion in cattle.

## Methods

### Sample collection

172 blood samples were collected from two dairy farms in Ecuador. 95 serum samples came from cattle that had aborted in the second and third trimester of their first pregnancy and 77 from cows of a randomly selected control group of the same age but without a history of abortion. For details, see Table 2. The cattle farms are located in Santo Domingo Province, a tropical area of the country. Both dairy cattle herds have about 2000 heads and blood samples were taken at random of approximately 40% of the cows that had aborted in the year 2018. Blood samples were taken between 2 and 10 months after the abortions had taken place. The herds are maintained as closed herds and in general the cows are artificially inseminated. Both farms have a relatively ¨normal¨ abortion rate of 3–5%.

**Table 2:** Number and average age with standard deviation of cows that aborted their first calves and aged-matched control cows from two cattle farms in Ecuador

	Cows that aborted and age	Control cows and age	
Farm 1 N_t_ = 93	*n* = 56, 35 ± 6 months	*n* = 37, 32 ± 7 months	
Farm 2 N_t_ = 79	*n* = 39, 65 ± 27 months	*n* = 40, 72 ± 33 months	
Nt = 172	n = 95	*N* = 77	

Blood was collected from the tail (coccygeal) vein in a red-top tube with serum clot activator. After clotting was complete, 1 ml of serum was collected and transferred into a cryovial tube and maintained at 4 °C until used in the ELISAs (max. 2 days).

### Serological testing

All serum samples were tested for antibodies against *C. burnetii, N. caninum*, and *B. abortus* by an enzyme-linked immunosorbent assay (ELISA) using commercial test kits and following the manufacturer’s instructions. The corresponding values for optical density were recorded by a 96-well microplates reader. Specific details of the ELISA kits, along with the sensitivities and specificities of the assays, are shown in Table 3.

**Table 3:** Commercially available ELISA test kits used for detecting antibodies against *B. abortus*, *N. caninum*, and *C. burnetii*. *The sensitivity (Se) and specificity (Sp) of the diagnostic kits were provided by the manufacturer of the kits

Infectious Agent	ELISA test kit	Manufacturer	Antigens	Se^*^	Sp^*^
*Brucella abortus*	ID Screen® Brucellosis Serum Indirect Multi-species	ID.vet Innovative Diagnostics, Grabels, France	LPS of *Brucella abortus*	100% (IC95%: 89.57–100%)	99.74% (IC95%: 99.24–99.91%)
*Neospora caninum*	ID Screen® *Neospora caninum* Indirect Multi-species	ID.vet Innovative Diagnostics, Grabels, France	Purified extract of *Neospora caninum*	100% (IC95%: 98.8–100%)	100% (IC95%: 99.41–100%),
*Coxiella burnetii*	ID Screen® Q fever indirect Multi-species	ID.vet Innovative Diagnostics, Grabels, France	phase I and phase II antigens *Coxiella burnetii*	100% (CI95%: 89.28–100%),	100% (CI95%: 97.75–100%)

### Statistical analysis

We performed a Chi-square test and a Fisher’s exact test analysis to compare the prevalence data of *Coxiella burnetii* infection and Neospora infection in cows that aborted vs. controls. In addition, we calculated odds ratios (ORs) with 95% confidence intervals (CIs). The statistical significance was set at *p* < 0.05. The data were analyzed using SPSS Version 22.0 for Windows.

## Additional file

Additional file 1: Detailed values for all serological tests for all the subjects included on this study. (XLSX 30 kb)

## Abbreviations

BHV: Bovine herpesvirus
BVD: Bovine viral diarrhea virus
IBR: Infectious Bovine Rhinotracheitis
OIE: World Organization of Animal Health
